# Development of a space cold atom clock

**DOI:** 10.1093/nsr/nwaa215

**Published:** 2020-08-31

**Authors:** Wei Ren, Tang Li, Qiuzhi Qu, Bin Wang, Lin Li, Desheng Lü, Weibiao Chen, Liang Liu

**Affiliations:** Key Laboratory of Quantum Optics and Center of Cold Atom Physics, Shanghai Institute of Optics and Fine Mechanics, Chinese Academy of Sciences, Shanghai 201800, China; Key Laboratory of Quantum Optics and Center of Cold Atom Physics, Shanghai Institute of Optics and Fine Mechanics, Chinese Academy of Sciences, Shanghai 201800, China; Key Laboratory of Quantum Optics and Center of Cold Atom Physics, Shanghai Institute of Optics and Fine Mechanics, Chinese Academy of Sciences, Shanghai 201800, China; Key Laboratory of Quantum Optics and Center of Cold Atom Physics, Shanghai Institute of Optics and Fine Mechanics, Chinese Academy of Sciences, Shanghai 201800, China; Key Laboratory of Quantum Optics and Center of Cold Atom Physics, Shanghai Institute of Optics and Fine Mechanics, Chinese Academy of Sciences, Shanghai 201800, China; Key Laboratory of Quantum Optics and Center of Cold Atom Physics, Shanghai Institute of Optics and Fine Mechanics, Chinese Academy of Sciences, Shanghai 201800, China; Research Center of Space Laser Information Technology, Shanghai Institute of Optics and Fine Mechanics, Chinese Academy of Sciences, Shanghai 201800, China; Key Laboratory of Quantum Optics and Center of Cold Atom Physics, Shanghai Institute of Optics and Fine Mechanics, Chinese Academy of Sciences, Shanghai 201800, China

**Keywords:** cold atom clock, in orbit, microgravity, fundamental physics

## Abstract

Atomic clocks with cold atoms play important roles in the field of fundamental physics as well as primary frequency standards. Operating such cold atom clocks in space paves the way for further exploration in fundamental physics, for example dark matter and general relativity. We developed a space cold atom clock (SCAC), which was launched into orbit with the Space Lab TG-2 in 2016. Before it deorbited with TG-2 in 2019, the SCAC had been working continuously for almost 3 years. During the period in orbit, many scientific experiments and engineering tests were performed. In this article, we summarize the principle, development and in-orbit results. These works provide the basis for construction of a space-borne time-frequency system in deep space.

## INTRODUCTION

Experiments in space provide new opportunities to test fundamental physics, and many great discoveries have been made recently. The space environment offers an ultra-high vacuum and infinite microgravity time at a large scale, which are not possible on ground. Recent progress in space science and technology allows us to design advanced space devices to test new physics, for example, the application of cold atoms in space will contribute to the construction of quantum sensors which have extremely high sensitivity [1], and can be of essential help to detect dark matter, gravitational wave, and to test the Standard Model [2].

Atoms can be cooled by laser beams to extremely low temperatures [3], and may be even further cooled by techniques such as evaporative cooling into a macroscopic quantum state such as the famous Bose-Einstein condensation [4,5]. On ground, such cold atoms are strongly affected by gravity not only in the cooling stage but also in the manipulation process, and therefore the physics for cold atoms in space has unique features to be explored.

Atomic clocks are becoming more and more important to modern society. Since their invention, the performance (stability and accuracy) of atomic clocks had been improved by 1 digit per 5 years until the mid-1990s when the traditional atomic clocks almost reached their limit. Thanks to the laser cooling technique, the performance of atomic clocks has been further improved with laser-cooled atoms, with which, not only is higher stability achieved because of a longer interrogation time, but also systematic uncertainties are reduced. Atomic clocks with laser-cooled atoms, so-called cold atom clocks, are now the most precise and accurate frequency standards, and are used in International Atomic Time (TAI).

A typical cold atom clock on ground is the atomic fountain clock [6,7]. Atoms are cooled and trapped in a magneto-optical trap at the bottom of a vacuum setup, then are launched upwards, and eventually fall downwards because of gravity after reaching their apogee. During the flight, the cold atoms interact twice with microwave field to yield Ramsey fringes, which are used to correct the microwave frequency. Such cold atom clocks have reached long-term stability at the 6 × 10^−17^ level [8], and are now widely used as the most accurate primary frequency standard.

Therefore, sending such a cold atom clock into space has potential applications in many fields. A space cold atom clock (SCAC) can improve the performance of GNSS (Global Navigation Satellite System), facilitate deep space navigation, and test the fundamental physics. However, although many efforts have been made [9–15], great challenges have impeded the deployment of such a SCAC.

We started a mission called Cold Atom Clock Experiment in Space (CACES) around the mid-2000s, which was approved by the China Manned Space Program (CMSP) in 2011. Since then, under the guidance of the CMSP, we developed a principle model, an engineering model and a flight model of a SCAC, which has been tested thoroughly on ground. The flight model was launched into orbit on 15 September 2016 with the Space Lab Tiangong-2 (TG-2), and was tested in orbit under the regulations of TG-2 until 19 July 2019 when the TG-2 deorbited. During almost 3 years in orbit, the SCAC was kept in operation and has carried out many tests and accumulated a large collection of data.

In this paper, we present the development and results of the SCAC, including the design, manufacture, and tests both on ground and in orbit. Firstly, we discuss the principles of operation and the fundamentals of design for SCAC. Then we describe the details on the engineering side and the basic function of major units, including the tests performed on ground. Moreover, we present the experimental results of in-orbit operation. Finally, we conclude the paper with an outlook towards the future.

## PRINCIPLE

There are two main differences between on ground and in-orbit scenarios for the operations of the cold atom clock. On ground, gravity drags cold atoms downwards, and thus the cold atom clock can be designed as a fountain, called the atomic fountain. In orbit, however, because of the microgravity environment where gravity is virtually absent, the cold atoms move in a uniform rectilinear line after they are launched, and thus the design of a fountain-type cold atom clock is not applicable for SCAC.

Another difference is the terrestrial magnetic field. Typically, an atomic fountain is set in a lab, where the terrestrial magnetic field is almost stable. It is relatively easy to shield the stable magnetic field. However, in orbit, the magnitude and direction of the terrestrial magnetic field changes during the aircraft's motion around the Earth, and automatic compensation of the magnetic field is required to keep any fluctuation in the interaction zone small.

**Figure 1.**
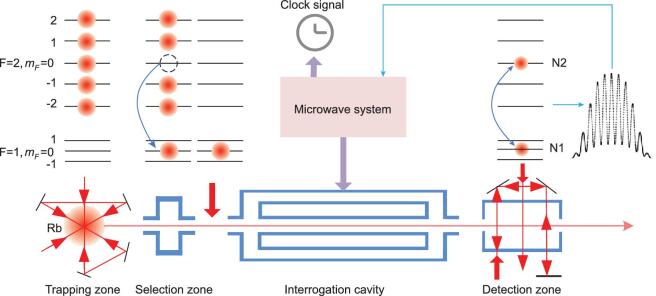
Principle and structure of the SCAC. The laser cooling and trapping zone is a MOT configuration with folded beam design. The ring interrogation cavity is used for the microwave field to interrogate cold atoms. In the detection zone, the cold atoms on both hyperfine states are detected. The clock signal is obtained by feeding the error signal to the microwave system.

The basic principle of a SCAC operating in orbit is shown in Fig. 1. Rubidium (^87^Rb) was chosen for our SCAC partly because of its relatively higher melting temperature, compared to cesium (^133^Cs), which reduces the design complexity of the atomic source for space application. Additionally, the collision shift of ^87^Rb is much smaller than that of ^133^Cs, and thus better performance can be expected with ^87^Rb [16]. The atoms are cooled and trapped in a magneto-optical trap (MOT), and then the cold atoms are launched by the moving optical molasses technique. During the launching stage, the cold atoms are further cooled by adiabatic cooling. Because of the microgravity, the cold atoms move in a straight line at a constant velocity. After state selection, the cold atoms are interrogated by the microwave field, and then detected by the laser excited atomic fluorescence.

The SCAC consists of four units: physics package, optical bench, microwave system and control electronics. The main part of the physical package is a titanium alloy vacuum tube whose vacuum is kept below 10^−7^ Pa. A ring cavity is used for the Ramsey interrogation of cold atoms by the microwave field. Three layers of Mu-metal are used to shield the magnetic field in the interrogation zone, while only one layer surrounds the cooling zone. The magnetic field in the interrogation zone is automatically compensated by regulating the current in the C-field coil through a servo loop, to keep the total magnetic field stable during motion of the spacecraft in orbit.

Together with a pair of anti-Helmholtz coils, two cooling laser beams are folded to form a compact MOT to reduce the power required for laser cooling. Because of the folded structure, all laser beams intersected in the MOT zone include the transmitting forward part and the backward part. Moreover, the frequencies of the two cooling beams can be controlled separately such that a moving optical molasses may be formed, and thus the cold atoms can be launched at a velocity of

(1) \begin{equation*} v = ({\omega _1} - {\omega _2})/k, \end{equation*}

where ${\omega _1}$, ${\omega _2}$ are the frequency of the two cooling laser beams, respectively, and $k$ is the wave vector. After being launched from MOT, the cold atoms are further cooled by the adiabatic cooling below the Doppler cooling limit.

The state selection is fulfilled by a combination of microwave excitation and laser pushing method. After the cooling and trapping stage, the atoms are concentrated at the state $|F = 2$〉 evenly distributed among five magnetic sub-states. The microwave power in the state selection cavity is adjusted such that the atoms at $|F = 2,\ {m_F} = \ 0$〉 are pumped to $|F = 1,\ {m_F} = \ 0$〉 at an efficiency of almost 100%. Then a laser beam pushes all other atoms at $|F = 2,\ {m_F} \ne 0$〉 states away, and the remaining atoms are used for microwave interrogation.

The Ramsey interrogation of a SCAC requires two cavities separated by a distance $D,$ corresponding to the interrogation time $T = D/v$ for cold atoms with velocity *v* in microgravity. The width of the central Ramsey fringe is directly related to the velocity as [17]

(2) \begin{equation*} \Delta \propto v/2D. \end{equation*}

Considering the dead time of the clock cycle combined with the size limitation, we made the choice of $D = 217{\rm{\ }}$mm for our SCAC.

**Figure 2.**
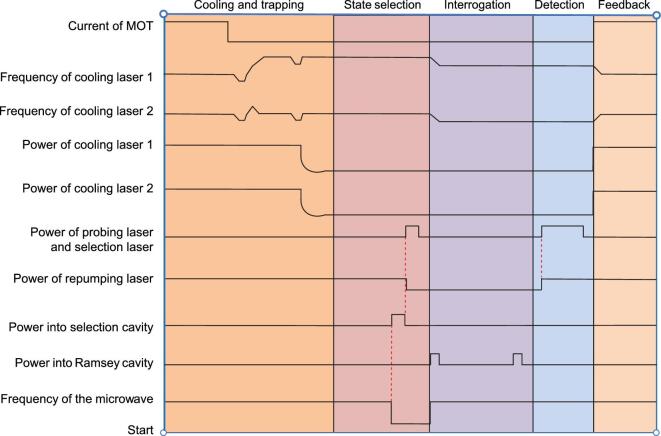
Timing sequence of SCAC.

The timing sequence of the SCAC is presented in Fig. 2. This process is composed of five stages: cooling and trapping, state selection, interrogation, detection and feedback. The power and frequency of cooling beams are regulated by the AOMs. Firstly, the rubidium atoms are captured and cooled to lower than 5 μK. Secondly, the cold rubidium atoms in state $|F = 2,\ {m_F} = \ 0$〉 are distinguished and selected into state $|F = 1,\ {m_F} = \ 0$〉 by a microwave field. Thirdly, the selected cold atoms are interrogated by a Ramsey separated oscillating field. Fourthly, the frequency error signal is obtained in the detection zone and is finally fed back to the microwave system.

## ENGINEERING OF SCAC

### Aims of the CACES mission

The European Space Agency (ESA) developed a cold atom clock, PHARAO, in their mission ACES, which aimed to operate a clock system in the International Space Station for the purpose of measuring the gravitational redshift, testing Lorentz invariance and searching variations of fundamental physical constants [12,14,15,18–20]. The CACES mission, under the support of the CMSP, is designed to operate a cold atom clock in the Space Laboratory TG-2. The main aims of this mission include:

- to test laser cooling of atoms in microgravity,
- to test the launching and the motion of cold atoms at very low velocity in microgravity,
- to test interrogation of cold atoms via Ramsey microwave fields in microgravity,
- to test the relevant key techniques in orbit, including the frequency stabilization of diode lasers, optical system, fiber-optic coupler, vacuum, temperature control, etc.,
- to estimate the performance of a SCAC in orbit,
- to accumulate data on the orbital environment for cold atom physics.

Meanwhile, the CACES mission also pioneers applications of space-borne quantum sensors based on cold atoms, such as the cold atom interferometer, cold atom gyroscope, optical clock, cold atom magnetometer, etc. Moreover, it will be helpful for future development of in-orbit precision measurement experiments for fundamental physics problems, such as detection of gravitational wave, the search for dark matter, etc.

### Principle model

Based on studies of the atomic fountain clock, a principle model of SCAC was built to test the main features of a cold atom clock for space application [21]. For the physics package, the principle model uses a ring structure as the Ramsey microwave cavity to simulate the operation of a cold atom clock in microgravity, which is different from an atomic fountain.

Besides the physics package, the optical bench is also an essential ingredient of a cold atom clock operated in orbit. A miniaturized optical bench was developed to test the stability of the most important optical components [22]. Several modules were developed and used in the bench. All units and modules were installed and fixed on an aluminum plate. The variance of output power did not exceed $ \pm 5{\rm{\% }}$ for 5 months, without any adjustment. The bench was employed for cooling, launching and detection of atoms in the SCAC for a long period. As demonstrated, the number of cooled atoms stayed relatively stable at a mean number $8.5 \times {10^6}$ with a variation of  $0.32 \times {10^6}$ for a long period, and the temperature of cooled atoms reached $1.5{\rm{\ \mu K}}$, which is almost the lowest temperature obtained from traditional three-dimensional laser cooling of ^87^Rb [23].

### Engineering model

Based on the design and tests of the principle model, from 2011 to 2013, an engineering model was developed and all the key engineering technologies were tested thoroughly.

#### Compact magneto-optical trap

**Figure 3.**
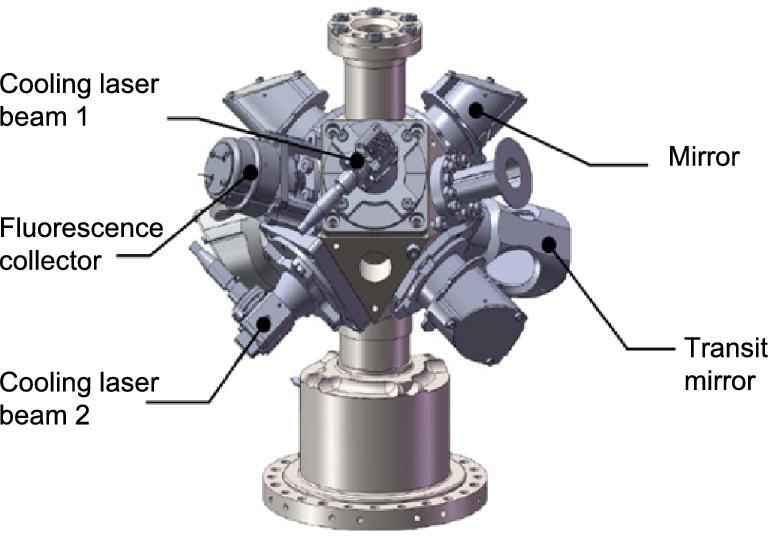
Set of the CMOT mechanical structure. Only two cooling laser beams are used instead of six.

A compact magneto-optical trap (CMOT) was designed and made for the engineering application of the space cold atom clock [24]. The schematic of the CMOT is shown in Fig. 3. Only two cooling laser beams were used, rather than the six used in conventional magneto-optical traps. Compared to a conventional trap, our CMOT has a series of advantages for space applications, especially for SCAC:

- more compact and efficient: a fold optical path scheme was adopted, and two cooling laser beams were input, which formed six cooling beams through transit mirrors. This design saves about 1/3 in volume and weight, and about 60% in laser power;
- more robust: no additional adjustment was required after the initial adjustment was completed. The whole mechanical structure is more stable and convenient for space applications.

The COMT passed thermal cycle tests and mechanical vibration and shock tests. It has been implemented in the engineering model and has operated for about 6 years in the laboratory, throughout which it maintained high performance without the need for realignment.

#### Ultra-high vacuum system

**Figure 4.**
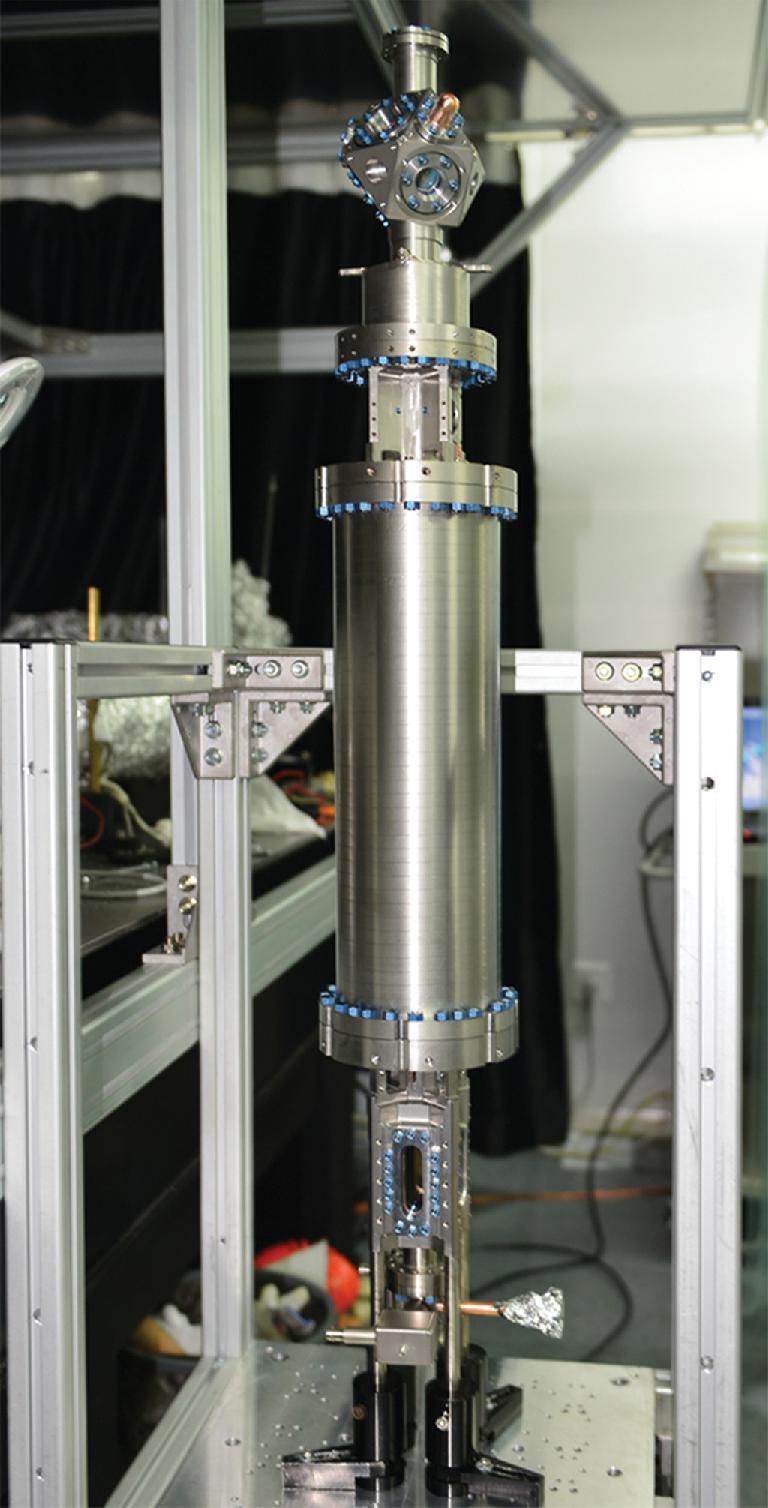
Ultra-high vacuum system used in the engineering model.

An ultra-high vacuum system was developed when the engineering model was manufactured [25] (Fig. 4). The whole system was made of titanium alloy material (Ti-6Al-4V), which is lightsome with high tensile strength and low thermal and electrical conductivity. Also, as this material is non-magnetic, it is especially suitable for manufacturing precision measurement instruments. The vacuum pump system was specially designed with four getters at each end of the interrogation cavity, which ensured that the ultra-high vacuum was maintained for a long time even without power supply. This feature can satisfy the requirements of space applications.

Also, from the engineering model of the ultra-high vacuum system, the distribution of the inside pressure was numerically calculated and tested. With these efforts, the vacuum performance of the SCAC was guaranteed.

#### Highly reliable optical bench

The optical system is a key part of a cold atom clock. For a SCAC, a compact and reliable optical bench is required [26,27]. For our optical bench, all the optical elements were integrated onto a 300 mm × 290 mm × 30 mm two-side optical table, with the result that the size and weight were greatly reduced compared with traditional optical systems. This optical bench passed all the space environmental qualification tests to guarantee high reliability.

In the SCAC, preparation and interrogation of the cold atoms are spatially separated and the transition probability of the rubidium atoms is measured by detecting the number of cold atoms on each hyperfine level via a resonance fluorescence method [27]. The photon emission rate fluctuation resulting from the optical frequency noise will affect the stability of SCAC during the detection process via Eq. (3).

(3) \begin{eqnarray*} \sigma_{y}^{c}\!\left( \tau \right) &=& \frac{1}{{2\pi {Q_{at}}}}\frac{1}{P}\sqrt {\frac{{{T_c}}}{\tau}} \mathop \int \nolimits_{{t_{det}}}^{} \sigma_{y}^{\gamma} \left[ {f\left( t \right)}\right.\nonumber\\ &&\left.{- f\left( {t - \Delta t} \right)} \right]dt,\end{eqnarray*}

where $\sigma _{y}^{\gamma} $ represents the photon emission rate fluctuation, which is related to the laser frequency noise. The subtraction of $f( t ) - f( {t - \Delta t} )$ represents a reduction of the laser frequency noise effect on the SCAC, which results from the separate detection of the atom number on the two levels. Consequently, a SCAC with a frequency stability of $\sim\!{10^{ - 13}}/ \sqrt \tau $ requires a laser frequency stability better than $\sim\!{10^{ - 11}}$ from 1 s to 10 000 s. The Allan deviation of laser frequency in the highly reliable optical system is of $\sim\!{10^{ - 11}}$ over averaging times from 1 to 10 000 s, and even falls into $\sim\!{10^{ - 12}}$ from 20 to 100 s, a contribution to the stability of SCAC less than the target level of $\sim\!{10^{ - 13}}/\sqrt \tau $.

#### Microwave system

**Figure 5.**
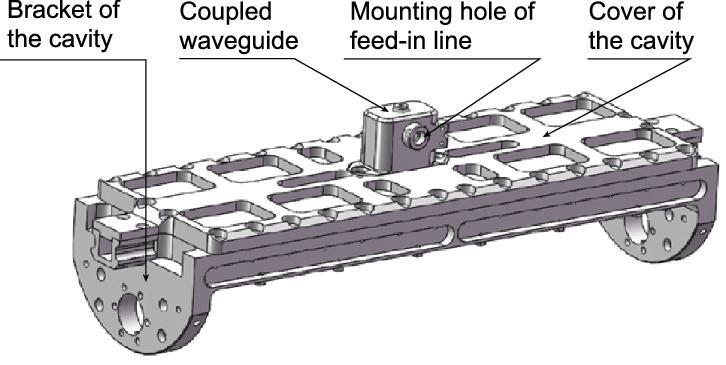
The ring microwave cavity for SCAC. This picture gives the assembly of the cavity shown in Fig. 1 of Ref. [28] in detail.

A microwave interrogation cavity in the SCAC is used to stimulate the clock transition of  ^87^Rb. Different from the fountain clock on ground, the SCAC’s Ramsey cavity must satisfy the conditions to operate in microgravity, in which the launched cold atoms move at constant velocity. To meet this demand, a dual-interaction zone interrogation cavity was developed [28]. The principle of the cavity is similar to that for cesium atoms in the ACES mission [12], but with different guidance structure. The cavity had a ring structure with four rectangular waveguide cavities connected end-to-end (Fig. 5), and was made of TC4 (Ti-6Al-4V) with silver coating. It had three major parts: bracket, cover and coupled waveguide. The microwave field is fed in from a hole on the coupled waveguide, and propagates in two opposite directions and finally forms a standing wave field, which was simulated and tested thoroughly, as discussed in Ref. [28].

The microwave system had two units, including a crystal oscillator which produced a 5 MHz signal, and a synthesizer which converted the 5 MHz signal to the transition frequency 6.834 GHz of the ^87^Rb. This was then fed into the microwave cavity for interrogation of cold atoms. A phase noise level of ${10^{ - 6.7}} \times {f^{ - 1.5}}$ rad^2^/Hz was realized. Such a microwave noise level guarantees the frequency stability of $1.4 \times {10^{ - 13}}{\tau ^{ - 1/2}}$ for SCAC operated in orbit [29].

#### Test of the engineering model

**Figure 6.**
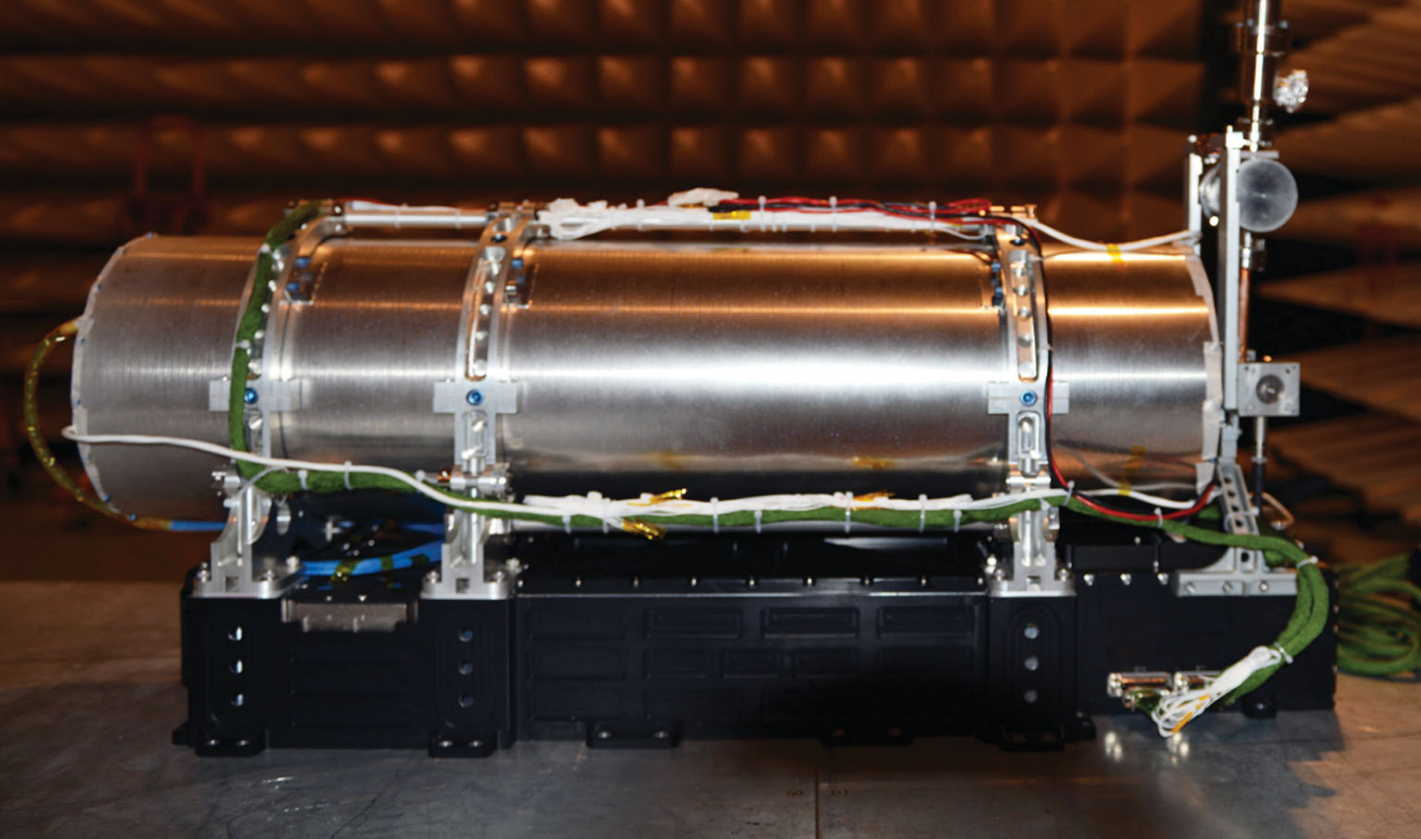
Photograph of the engineering model for SCAC.

The engineering model of SCAC was manufactured and assembled as shown in Fig. 6. After integration, this model was tested on ground carefully [30]. The engineering model had a good medium-term frequency stability of 1.5 × 10^−14^@1000 s. The tests and results provided a basis for development of the flight model.

### Flight model

**Figure 7.**
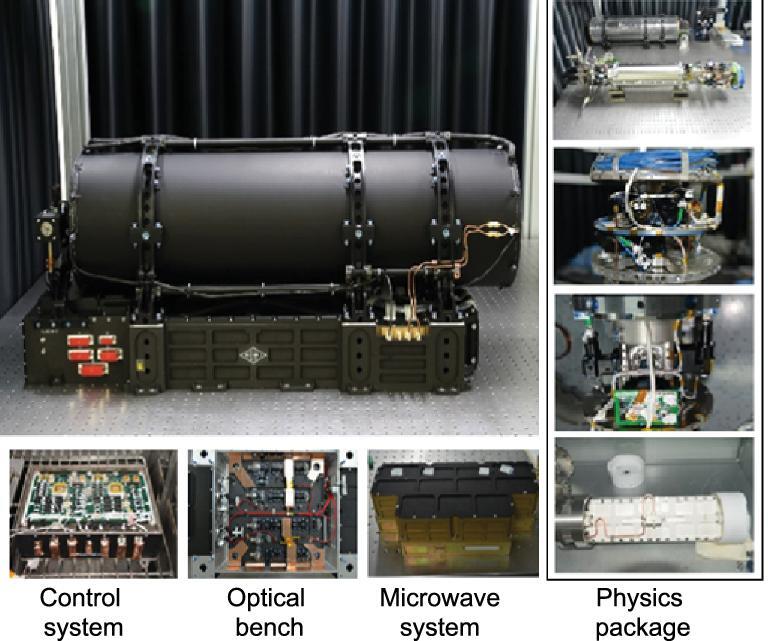
Photograph of the flight model for SCAC, which contains more details compared to Supplementary Fig. 1 of Ref. [32].

After the success of the engineering model, from 2013 to 2016, a flight model was developed for the flight mission of TG-2 (Fig. 7). An automatic compensation device was added to the flight model to keep the magnetic field constant inside the physical package during the flight in the orbit around the Earth [31].

The thermal and mechanical tests were carried out according to the regulations of the CMSP. The performance of this model was also tested under different conditions and met the requirements for space application. About 10^8^ rubidium atoms can be captured and their temperature can be lower than 5 μK in the flight model's ground tests. The Ramsey fringe was obtained with a full-width-at-half-maximum (FWHM) smaller than 10 Hz in laboratory and the frequency stability was obtained by comparing the flight model against an H-Maser even though the performance of the SCAC was degraded as a result of the gravity on ground. These results satisfy the demands of the CACES project.

The flight model was handed over to the CMSP in May 2016 and went through tests together with all the payloads of TG-2 under unified coordination in

June 2016. After the combined tests, the flight model was then installed into the spacecraft in September 2016.

## TEST IN ORBIT

The TG-2 was launched on 15 September 2016 at the Jiuquan Satellite Launch Center, and the SCAC was switched on during the second day, and started almost immediately until the time that TG-2 deorbited on 19 July 2019. The in-orbit tests involved validation of the engineering objectives and exploration of in-orbit scientific phenomena.

**Figure 8.**
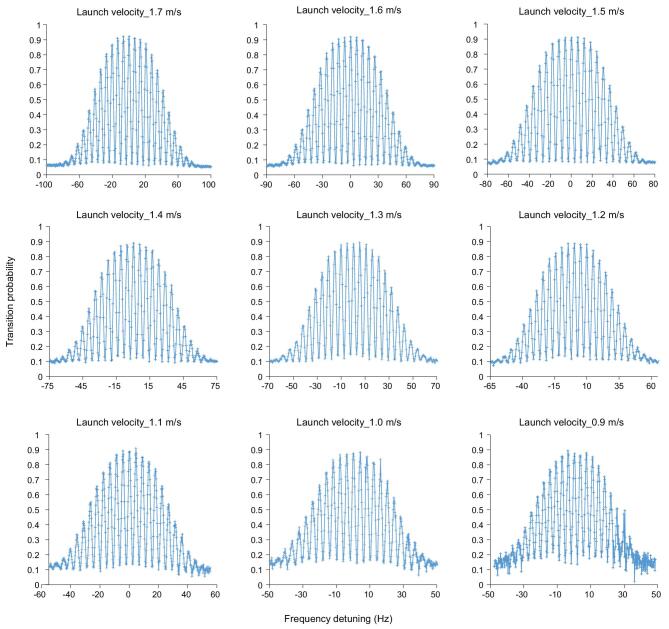
Ramsey fringes versus frequency detuning with launch velocities of different cold atoms.

With the flight model in orbit, time-of-flight signals were detected in the detection region of the SCAC, and some typical and expected Ramsey fringes were obtained (Fig. 8). The relation between the FWHM of central Ramsey fringe and the atoms’ launch velocity was determined, which is consistent with the calculated results [29,32]. The feed-into microwave power was also determined from the test, and agreed well with the calculated results. The signal-to-noise ratio of the SCAC was measured at the launching velocities of different atoms, from which the in-orbit short-term frequency stability of the SCAC was estimated to be close to $3.0 \times {10^{{\rm{ - }}13}}{\tau ^{{\rm{ - 1/2}}}}$ with a clock period of 2.0 s, as shown in Ref. [32]. Also, a closed-loop operation was carried out by feeding the error signal to the direct digital synthesizer (DDS) of the microwave system to verify the in-orbit long-term operation mechanism [32]. This is particularly useful for the design of the next generation cold atom clock, which is expected to operate in the Chinese Space Station in the future.

## OUTLOOK

The CACES mission fulfilled all its goals before the deorbit of TG-2. The engineering model, as the backup of the flight model, is still working in the laboratory for continuous testing. The successful operation of a SCAC in orbit paves the way for wide applications of cold atom technologies in space, such as the cold atom interferometer, cold atom gyroscope, cold atom optical clock, etc. Quantum sensors based on cold atoms will play a more and more important role in space fundamental physics [1].

Based on the success of the CACES mission, in the Chinese Space Station, in-orbit experiments on the cold atom clock will be carried out based on the ultra-high precision time-frequency cabinet and the ultra-cold atom physics cabinet, which are key adopted technologies of the SCAC.

With all the works above, it is expected that time-frequency synchronization between ground and space will be achieved, opening a new door within deep space exploration and basic physics research.
